# Failure of PCR to Detect *Treponema pallidum* ssp. *pertenue* DNA in Blood in Latent Yaws

**DOI:** 10.1371/journal.pntd.0003905

**Published:** 2015-06-30

**Authors:** Michael Marks, Samantha Katz, Kai-Hua Chi, Ventis Vahi, Yongcheng Sun, David C. Mabey, Anthony W. Solomon, Cheng Y. Chen, Allan Pillay

**Affiliations:** 1 Clinical Research Department, Faculty of Infectious and Tropical Diseases, London School of Hygiene & Tropical Medicine, London, United Kingdom; 2 The Hospital for Tropical Diseases, London, United Kingdom; 3 Laboratory Reference and Research Branch, Division of STD Prevention, National Center for HIV/AIDS, Viral Hepatitis, STD and Tuberculosis Prevention, Centers for Disease Control and Prevention, Atlanta, Georgia, United States of America; 4 Ministry of Health and Medical Services, Honiara, Solomon Islands; University of Tennessee, UNITED STATES

## Abstract

Yaws, caused by *Treponema pallidum* ssp. *pertenue*, is a neglected tropical disease closely related to venereal syphilis and is targeted for eradication by 2020. Latent yaws represents a diagnostic challenge, and current tools cannot adequately distinguish between individuals with true latent infection and individuals who are serofast following successful treatment. PCR on blood has previously been shown to detect *T*. *pallidum* DNA in patients with syphilis, suggesting that this approach may be of value in yaws. We performed real-time PCR for *Treponema pallidum* ssp. *pertenue* on blood samples from 140 children with positive *T*. *pallidum* Particle Agglutination (TPPA) and Rapid Plasma Reagin (RPR) tests and 7 controls (negative serology), all collected as part of a prospective study of yaws in the Solomon Islands. All samples were also tested by a nested PCR for *T*. *pallidum*. 12 patients had clinical evidence of active yaws whilst 128 were considered to have latent yaws. 43 children had high titre rapid plasma reagins (RPRs) of ≥1:32. PCR testing with both assays gave negative results in all cases. It is possible that the failure to detect *T*. *pallidum* ssp. *pertenue* in blood reflects lower loads of organism in latent yaws compared to those in latent infection with *T*. *pallidum* ssp. *pertenue*, and/or a lower propensity for haematogenous dissemination in yaws than in syphilis. As the goal of the yaws control programme is eradication, a tool that can differentiate true latent infection from individuals who are serofast would be of value; however, PCR of blood is not that tool.

## Author Summary

Yaws is a bacterial infection closely related to syphilis. The WHO has launched a worldwide campaign to eradicate yaws by 2020. For each clinically apparent case, many close contacts are infected but do not show clinical signs, which is called latent yaws. Currently, diagnosis for these patients relies on the detection of antibodies using syphilis serology. Reliance on detection of antibodies for diagnosis of latent yaws is problematic as the test may remain positive with a low titre despite successful treatment. This makes the interpretation of surveillance data in a post-mass treatment setting difficult for programme managers. In syphilis, PCR can detect *T*. *pallidum* ssp. *pallidum* in the blood of patients with latent disease. We used similar techniques to try and detect the yaws bacterium in the blood of patients seen as part of a study of yaws in the Solomon Islands. Although many people had positive antibodies for yaws consistent with latent yaws, we were unable to detect the bacterium in the blood. This may reflect the lower virulence of the yaws bacterium compared to syphilis. Currently available molecular techniques are therefore not able to aid programme managers in conducting post-mass drug administration surveillance for latent yaws. The development of alternative diagnostic tests should be considered.

## Introduction

Yaws, caused by infection with *Treponema pallidum* ssp. *pertenue*[1], is targeted for eradication by 2020[2]. The organism is closely related to *Treponema pallidum* ssp. *pallidum*, the causative agent of venereal syphilis. The mainstay of the World Health Organization (WHO) eradication strategy is mass drug administration (MDA) of azithromycin in endemic communities. A significant concern for the implementation of this strategy is the development of resistance to azithromycin. Two mutations in the 23s rRNA gene have been associated with azithromycin resistance and treatment failure in syphilis[3]. Resistance to azithromycin and treatment failure are now seen frequently in syphilis in a number of regions, including North America, Europe, Asia and Australasia[4–6], but have not been observed in low income settings where use of macrolide antibiotics for other diseases is less common[7].

As in venereal syphilis, latent infection also occurs in yaws. There are estimated to be 5–6 latently infected cases for each case of active disease, and these individuals may relapse for up to 5 years[8,9]. There is a theoretical risk that azithromycin may be less effective in treating latent infection because the bacterium is less metabolically active.

A second related issue in latent yaws is the inability to differentiate between individuals with latent yaws who are at risk of relapse, and individuals who have been successfully treated but remain sero-positive. The gold-standard test for latent infection is the Rabbit-Infectivity Test (RIT), but availability of this technique is limited to highly specialized research laboratories[10]. PCR has been shown to have a diagnostic yield of up to 40% in latent syphilis, depending on the sample used[11]. The diagnosis of latent yaws is further complicated by the finding that other organisms may be responsible for ulcers in individuals seropositive for yaws[12]. The development of a molecular technique to detect *T*. *pallidum* ssp. *pertenue* would allow the differentiation of true latent yaws from individuals with serofast status, and might also facilitate the detection of azithromycin resistance in latent yaws if it were to appear.

The aim of this study was to assess whether it is possible to use nested PCR and/or real-time PCR to detect *T*. *pallidum* ssp. *pertenue* DNA in the blood of individuals with latent yaws.

## Methods

Samples were collected as part of a large prospective study of the epidemiology of yaws in the Solomon Islands. The survey methodology has been described elsewhere[13]. Briefly, we recruited a total of 1,497 children from 30 randomly-selected households in each of 25 randomly selected clusters in each Western and Choiseul provinces of the Solomon Islands, collected information on symptoms and treatment of yaws, and performed a standardised examination of the skin. Venepuncture was performed for collection of both a serum sample for serological testing and a whole blood sample; the latter was stored in a PAXgene DNA tube (Qiagen Inc., Valencia, CA), which maintain DNA integrity for up to 2 weeks at ambient temperature. Samples were transferred to Honiara National Referral Hospital within 5 days and stored at -20°C. Serum samples were shipped on dry ice to London School of Hygiene and Tropical Medicine (LSHTM). Whole blood samples were shipped on dry ice to the Centers for Disease Control and Prevention (CDC). For this study, paired sera and PAXgene blood samples from 147 children (9.8% of total study population) were used for serology and PCR analysis.

Serum samples were tested by *Treponema pallidum* particle agglutination (TPPA; Mast Diagnostics, Merseyside, UK). In individuals with a positive TPPA, a quantitative rapid plasma reagin test (RPR; Deben Diagnostics, Ipswich, UK) was performed.

DNA was manually extracted from 1–1.2ml PAXgene samples at CDC, using the QIAamp DNA Blood Midi Kit (Qiagen Inc.) and tested in a real-time quadriplex PCR containing primers and TaqMan probes targeting the *tp0858* gene, two areas of the *tprI* (*tp0620*) gene and the human RNase P gene to monitor for PCR inhibition [14]. This PCR has been reported to successfully identify *T*. *pallidum* ssp. *pertenue* from skin lesions of primary and secondary yaws and differentiate it from the other two subspecies. The quadriplex PCR amplification was performed as described previously [14]. Briefly, 10–20 μL of DNA was used in a reaction containing a final concentration of 1x PerfeCTa MultiPlex qPCR SuperMix (Quanta Biosciences Inc., Gaithersburg, MD). All reactions were performed using the Rotor-Gene Q real-time PCR instrument (Qiagen Inc.) with the following conditions: initial hold at 95°C for 4 min followed by 50 cycles of 95°C for 20 sec and 60°C for 1 min. A no-template control and custom synthesized DNA fragments with unique genetic signatures in *tp0858* or *tprI* (insert size is ~250 to 400 bp) cloned into pIDTSmart plasmids (Integrated DNA Technologies, Coralville, IA) were used as positive controls. In addition, each run also included genomic DNA from a lesion sample that previously tested positive for *T*. *pallidum* ssp. *pertenue*.

A nested PCR consisting of outer primers for a conventional PCR and inner primers for a real-time PCR was used to specifically amplify one of two alleles of the 23S rRNA gene (*tp0226*) of *T*. *pallidum* ssp. *pertenue*. The outer PCR which amplifies a 1602-bp fragment was performed using a final concentration of 200 nM each of the sense (5’–GTACCGCAAACCGACACAG) and antisense (5’–GCGCGAACACCTCTTTTTAC) primers[4]. Reactions were carried out in a 50 μL volume containing 200 μM of dNTPs, 1x PCR buffer, 1.25 U of Takara Ex Taq polymerase (Clontech Laboratories, Inc., Mountain View, CA), and 20 μL of DNA in an Applied Biosystems GeneAmp PCR system using the following conditions: initial hold at 94°C for 3 min followed by 45 cycles of 94°C for 1 min, 63°C for 1 min, and 68°C for 2 min, with a final extension cycle at 68°C for 10 min. A no-template control and purified genomic DNAs from *T*. *pallidum* ssp. *pallidum* strain Nichols and *T*. *pallidum* ssp. *pertenue* strain CDC2575 were included in each run. The inner real-time PCR which amplifies a 185-bp fragment of the outer PCR amplicon above was performed using primers and TaqMan probes as described previously [3]. Briefly, 1 μL of outer PCR amplicon was used in a 25 μL reaction containing a final concentration of 1x PerfeCTa MultiPlex qPCR SuperMix. All reactions were performed using the Rotor-Gene Q real-time PCR instrument with the following conditions: initial hold at 95°C for 4 min followed by 50 cycles of 95°C for 20 s and 60°C for 1 min.

The real-time PCR controls used were the same as those for conventional PCR above. Blood samples were defined as being PCR-negative for *T*. *pallidum* ssp. *pertenue* if an amplification curve or a cycle threshold (Ct) value was not observed after real-time PCR, provided all positive control DNA samples gave the expected results. The analytical sensitivities of the real-time PCR and nested PCR were determined by testing a 10-fold serial dilution of purified genomic DNA from the *T*. *pallidum* Nichols strain.

As the yield of PCR positivity on blood was uncertain we pragmatically elected to test all seropositive samples with a dual TPPA and RPR (titer ≥1/4–128) positivity, and a small number of dual positives with an RPR titer <1/4 and seronegative controls. We deliberately over-represented individuals with high titre positive serology as these individuals are more likely to be truly infected with *T*. *pallidum* ssp. *pertenue* rather than having serofast serology.

Individuals were considered to have latent yaws if they had reactive serology with no current clinical manifestations of active disease, and active yaws if they had skin lesions consistent with yaws and reactive serology. For the purposes of this analysis, we defined a low titre RPR result as an RPR of <1/32 and a high titre RPR result as an RPR of ≥1/32. All analyses were performed in Stata 13.1 (Statacorp, Texas).

Written informed consent was obtained from the head of each household, who was the parent or guardian of children enrolled in the study, and assent was obtained from all children. Ethical approval for the study was granted by the ethics committees of the Ministry of Health and Medical Services in the Solomon Islands, LSHTM in the UK, and the CDC in the US.

## Results

Of the 1,497 children that were enrolled in the baseline survey, paired sera and PAXgene blood samples from 147 children were tested [13]. The median age of children included was 9 years (IQR 7–12) and 90 (61%) were male. 12 (8.2%) children had reactive serology (reactive RPR and TPPA) and a skin lesion consistent with active yaws, and 128 (87%) had reactive serology without evidence of active disease and were classified as latent yaws. The remaining 7 (5%) children had non-reactive serology, and their samples were included as negative controls for PCR (Supplemental Data 1).

The prevalence of active yaws in the population included here was significantly higher than in the overall baseline survey population (3.7%, p = 0.012)[13], consistent with our deliberate oversampling for this study of individuals with reactive serology. Of individuals included 97 children (66%) had a low titre RPR, and 43 children (29%) had a high titre RPR.

All blood samples tested negative for *T*. *pallidum* ssp. *pertenue* with the real-time quadriplex PCR. Nested-PCR amplification also was negative for all samples. The internal control (human RNase P gene) was amplifiable from all blood samples. The limit of detection (LOD) of the real-time PCR was determined to be approximately10 to 100 genomic copies of the *T*. *pallidum* Nichols strain per reaction. The real-time PCR had an efficiency of 90% and a coefficient of correlation (R^2^) of 0.98. The nested PCR products that were derived from serial 10-fold dilutions all had very low threshold cycle (Ct) values as compared to the non-nested real-time PCR assay, but the LOD remained the same. Although the LOD of both assays was 10 to 100 genomic copies, both assays did occasionally detect samples with <10 genomic copies.

## Discussion

In this study we were unable to detect *T*. *pallidum* ssp. *pertenue* DNA in the blood of patients with latent or clinical yaws infection who were diagnosed on the basis of dual seropositivity. We extracted DNA from a larger volume of blood (1–1.2mL) compared to studies of syphilis where ≤ 500 μL blood was used[15,16], which might have been expected to increase the diagnostic yield. By contrast, *T*. *pallidum* DNA has been detected in up to 40% of individuals with latent syphilis [11]. The real-time quadriplex PCR we used initially has a detection limit of 10 to 100 genomic copies[14]. Subsequent testing with a real-time PCR modified into a nested PCR was also negative in all the samples, suggesting that a lack of sensitivity of the real-time quadriplex PCR does not explain our findings. In addition, amplification of the human DNA control in all samples ruled out the possibility of PCR inhibitors and showed that the integrity of the DNA was preserved. Nested PCR is presumed to be more sensitive than real-time PCR although there is limited published data to support this. The nested-PCR assay and real-time PCR we tested had the same detection limit; however, it is possible that nested PCR might detect *T*. *pallidum* DNA more frequently in samples with <10 genomic copies as suggested by the low Ct values versus real-time PCR but this remains to be investigated.

Haematogenous dissemination of spirochaetes is thought to be responsible for the development of late stage disease in treponemal infection, suggesting that patients are at least temporarily spirochaetaemic. In fact, previous studies have shown that PCR can be used to detect treponemal DNA from the blood at every stage of syphilis[16]. Severe late stage cardiovascular and neurological manifestations are well recognised in untreated syphilis. Whilst some studies have suggested that neurological and cardiovascular manifestations may rarely occur in yaws[17], these findings are difficult to interpret given the historical difficulty in differentiating *T*. *pallidum* ssp. *pallidum* and *T*. *pallidum* ssp. pertenue. It is possible that compared to *T*. *pallidum* ssp. *pertenue*, *T*. *pallidum* ssp. *pallidum* is more virulent and associated with more frequent spirochaetaemia, and that our results reflect this difference.

In our study we used a clinical and serological definition of yaws. It is increasingly recognised that other organisms may cause ulcers in individuals in yaws endemic communities[12,18]. Whilst this might have resulted in some individuals who truly had latent yaws as being classified as having active disease it does not alter our finding that we were unable to detect *T*. *pallidum* ssp. *pertenue* from any of the individuals in this study. An attenuated clinical phenotype of yaws has been described in the Solomon Islands with little advanced tertiary disease seen [19]. Although the cause of attenuated disease is not known, it might be possible that the strain of *T*. *pallidum* ssp. *pertenue* in the Solomon Islands is less invasive than that seen in other countries such as Ghana where late stage disease is still seen. Studies should be conducted in other countries where yaws is endemic to confirm our findings.

Our findings have implications for surveillance strategies in the WHO yaws eradication campaign. Relapses of latent disease could be responsible for onward transmission to previously uninfected contacts, but current tools can not differentiate individuals who are serofast from individuals with true latent infection and who therefore require treatment. As the goal of the yaws control programme is eradication, a tool that could differentiate true latent infection from individuals who are serofast would be of value in a post-MDA setting, where clinical and serological surveys are recommended to identify individuals who require re-treatment. Our findings suggest that PCR of blood is unlikely to be useful in that context.

Whilst azithromycin resistance has not yet been reported in *T*. *pallidum* ssp. *pertenue*, it is now well-established in *T*. *pallidum* ssp. *pallidum* and monitoring for its emergence will be important to yaws programmes in the post-MDA phase. We had hoped that PCR of blood samples would allow monitoring for azithromycin resistance in individuals with latent yaws. As we were not able to amplify *T*. *pallidum* ssp. *pertenue* DNA from blood samples, we were not able to apply PCR testing for mutations associated with azithromycin resistance.

In conclusion, we were unable to detect *T*. *pallidum* ssp. *pertenue* DNA in the blood of individuals with latent yaws. The development of improved diagnostic tools should be a high priority for the yaws eradication campaign.

## Supporting Information

S1 Checklist STROBE checklist. (DOC)

S1 Dataset Study dataset. (XLS)
